# Handmade Cloned Transgenic Sheep Rich in Omega-3 Fatty Acids

**DOI:** 10.1371/journal.pone.0055941

**Published:** 2013-02-20

**Authors:** Peng Zhang, Peng Liu, Hongwei Dou, Lei Chen, Longxin Chen, Lin Lin, Pingping Tan, Gabor Vajta, Jianfeng Gao, Yutao Du, Runlin Z. Ma

**Affiliations:** 1 State Key Laboratory of Molecular and Developmental Biology, Institute of Genetics and Developmental Biology, Chinese Academy of Sciences, Beijing, China; 2 BGI ARK Biotechnology Co., Ltd, Shenzhen, China; 3 IRIS, Central Queensland University, Rockhampton, Australia; 4 School of Life Sciences, Shihezi University, Shihezi, China; 5 BGI-Shenzhen, Shenzhen, China; 6 Graduate University of the Chinese Academy of Sciences, Beijing, China; Justus-Liebig-Universität, Germany

## Abstract

Technology of somatic cell nuclear transfer (SCNT) has been adapted worldwide to generate transgenic animals, although the traditional procedure relies largely on instrumental micromanipulation. In this study, we used the modified handmade cloning (HMC) established in cattle and pig to produce transgenic sheep with elevated levels of omega-3 (n−3) fatty acids. Codon-optimized nematode *mfat-1* was inserted into a eukaryotic expression vector and was transferred into the genome of primary ovine fibroblast cells from a male Chinese merino sheep. Reverse transcriptase PCR, gas chromatography, and chromosome analyses were performed to select nuclear donor cells capable of converting omega-6 (n−6) into n−3 fatty acids. Blastocysts developed after 7 days of *in vitro* culture were surgically transplanted into the uterus of female ovine recipients of a local sheep breed in Xinjiang. For the HMC, approximately 8.9% (n  = 925) of reconstructed embryos developed to the blastocyst stage. Four recipients became pregnant after 53 blastocysts were transplanted into 29 naturally cycling females, and a total of 3 live transgenic lambs were produced. Detailed analyses on one of the transgenic lambs revealed a single integration of the modified nematode *mfat-1* gene at sheep chromosome 5. The transgenic sheep expressed functional n−3 fatty acid desaturase, accompanied by more than 2-folds reduction of n−6/n−3 ratio in the muscle (*p*<0.01) and other major organs/tissues (*p*<0.05). To our knowledge, this is the first report of transgenic sheep produced by the HMC. Compared to the traditional SCNT method, HMC showed an equivalent efficiency but proved cheaper and easier in operation.

## Introduction

Sheep is one of the most important domestic animal species for human consumption of meat protein and milk. With new knowledge and understanding that a number of human diseases can be effectively prevented via improved and balanced nutrition, the nutritional value of sheep meat and milk could be further increased by elevated levels of polyunsaturated fatty acids (omega-3 or n−3 PUFAs). Omega-3 is an essential nutrient for human and has been demonstrated to have preventive and therapeutic effects on certain diseases of cardiovascular nature, arthritis, cancer, as well as neuropathic problems [1]–[4]. Unfortunately, supply of omega-3 in human is totally dependent on dietary intake due to our body’s inability to synthesize the essential nutrients. While the exact reason on why human and most other mammals have lost their abilities to synthesize omega-3 during the evolution remains unclear [5], certain primary organisms like *C. elegans* can efficiently covert n−6 into n−3 PUFAs [6]–[8]. It would be ideal to produce sufficient amount of omega-3 in domestic animals via genetic engineering, which would be much better than capturing and killing of deep-sea fishes for the same essential nutrient. In this regard, transgenic pigs and cattle rich in n−3 fatty acids were generated to explore the possibility [2], [9].

Technology of somatic cell nuclear transfer (SCNT) has been adapted worldwide since the successful generation of the sheep “Dolly” [10]. However, the traditional SCNT procedures rely on specialized instruments of micromanipulation, which are expensive and technologically demanding. An alternative technique termed handmade cloning (HMC), established previously [11], has proved to be efficient for animal cloning in several domestic species [12]–[17]. For the procedure of HMC, in contrast to traditional SCNT, the nucleus of an oocyte is removed manually by slicing off a portion of the zona-free oocyte with a sharp microblade, and a somatic nucleus is introduced by fusion of two enucleated oocytes with the somatic cell under an ordinary light microscope.

In this study, we aimed to establish a reliable and robust HMC procedure to generate transgenic sheep with increased levels of omega-3. We report here that a single copy of nematode *mfat-1* gene was successfully integrated into the sheep *Cep120* genomic locus on chromosome 5. Both of the introduced *mfat-1* and the host gene *Cep120* were functional, and three live births of the transgenic sheep were produced by the HMC. Tissue examination of one of the three new born transgenic lambs showed that the introduced *mfat-1* effectively lowered the n−6/n−3 ratio in the muscle and other major organs/tissues. If the nematode *mfat-1* gene is finally proved to be heritable and functionally stable in the host genome, the transgenic animals would potentially contribute better to human health for evaluated levels of omega-3 in meat and milk.

## Results and Discussion

**Figure 1:**
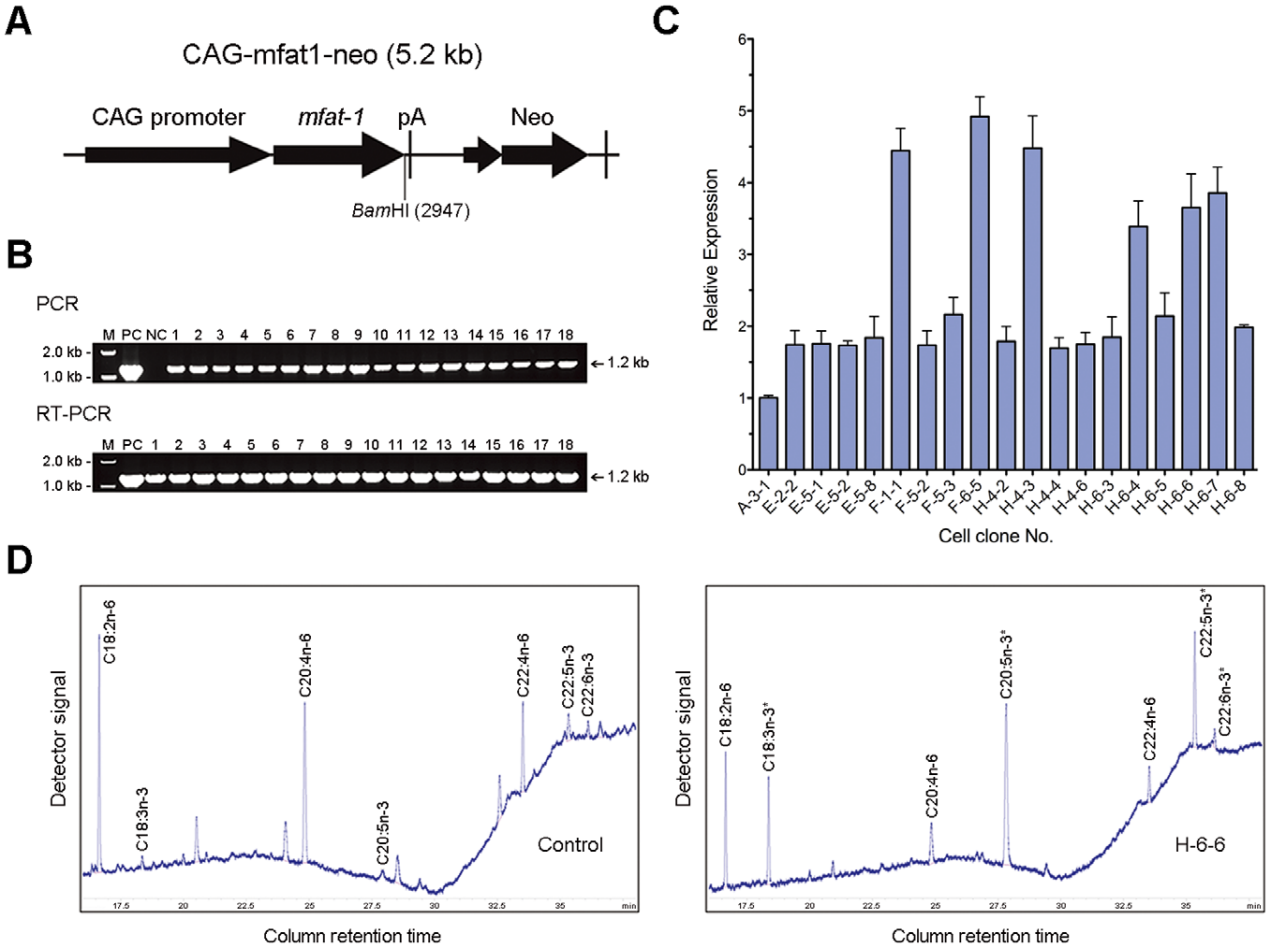
Establishment and analysis of transgenic clonal donor cells. (**A**) Schematic representation of n−3 fatty acid desaturase gene with linearized expression vectors. (**B**) Detection of the *mfat-1* gene in Geneticin-resistant cell clones by PCR and RT-qPCR. *mfat-1* expression vector was used as the template for positive control (PC) and untransfected syngenic cells was used as the negative control (NC). (**C**) Quantitative PCR analysis of *mfat-1* expression in positive cell clones. cDNA representing *mfat-1* was amplified with sequence specific primers. The beta-actin was used as internal control and the expression level observed in the transgenic donor cell was normalized to the value of A-3-1. (**D**) Partial gas chromatograph traces showing the polyunsaturated fatty acid profiles of total cellular lipids from the H-6-6 cells and the control cells. Note the level of n-6 polyunsaturated acids are lower whereas n−3 fatty acids are abundant in the *mfat-1* cells (right) as compared with the control cells (left), in which there is very little n−3 fatty acid.

Following the HMC procedure, we successfully generated 3 transgenic sheep that carry and express the nematode *mfat-1* gene. The coding sequence of nematode *fat-1* (1209 bps) was modified for optimal expression in mammalian system (*mfat-1*) prior integrating to the primary fibroblast cells of the Chinese merino sheep (Figure 1 A, B). Recombinant cells were screened by PCR and RT-qPCR for the presence and level of *mfat-1* mRNA expression (Figure 1C), and the cellular fatty acids composition was assessed by gas chromatography for *mfat-1* function (Figure 1D). In the recombinant clonal cells expressing *mfat-1*, n−6 fatty acids (18∶2n−6, 20∶4n−6, and 22∶4n−6) were successfully converted to the corresponding n−3 (18∶3n−3, 20∶5n−3, and 22∶5n−3), and the ratio of n−6/n−3 were reduced from 8.60∶1 to 0.45∶1, a change of more than 19-folds of reduction compared to the non-transfected cells (Figure 1D, Table 1). The candidate clones selected (F-1-1, F-6-5, and H-6-6) showed the normal karyotype (data not shown). We eventually selected the clone H-6-6 for the subsequent HMC procedure; partially due to a relatively higher ratio of n−6/n−3 conversion was observed in that clone (Table 1).

**Table 1:** Composition and ratio of n−6 and n−3 PUFA in the syngenic control and the transgenic cells expressing *mfat-1* gene.

Fatty acids	Control Cells	*mfat-1* Cells		
		F-1-1	F-6-5	H-6-6
LA (18∶2n−6)	6.35±0.48	4.85±0.03**	5.01±0.13**	4.57±0.19**
ALA (18∶3n−3)	0.06±0.05	1.61±0.13**	1.32±0.16**	3.92±0.45**
AA (20∶4n−6)	5.90±1.06	4.06±0.50*	5.62±0.14	2.64±0.38**
EPA (20∶5n−3)	0.00	6.24±0.06**	4.24±0.06**	10.54±0.18**
ADA (22∶4n−6)	2.47±0.39	1.86±0.36	1.83±0.30*	1.73±0.29*
DPA (22∶5n−3)	1.11±0.18	3.61±0.16**	1.55±0.18*	4.72±0.26**
DHA (22∶6n−3)	0.54±0.12	0.76±0.06*	0.43±0.22	0.71±0.06
Total n−6	14.72±1.90	10.76±0.84*	12.49±0.65	8.95±0.53**
Total n−3	1.71±0.08	12.22±0.35**	7.54±0.55**	19.94±0.65**
**n**−**6/n**−**3 ratio**	8.60±1.50	0.88±0.09**	1.65±0.16**	0.45±0.04**

Fatty acids composition is presented as a percentage of the total cellular lipids from the control cells and *mfat-1* cells. Each value represented the mean ± standard deviation from three cell samples in each group with two independent measurements for each sample.

a Statistical analysis using the two tailed student *t*-test. Significant differences between the syngenic control and *mfat-1* cells were marked (*P<0.05; **P<0.01).

**Figure 2:**
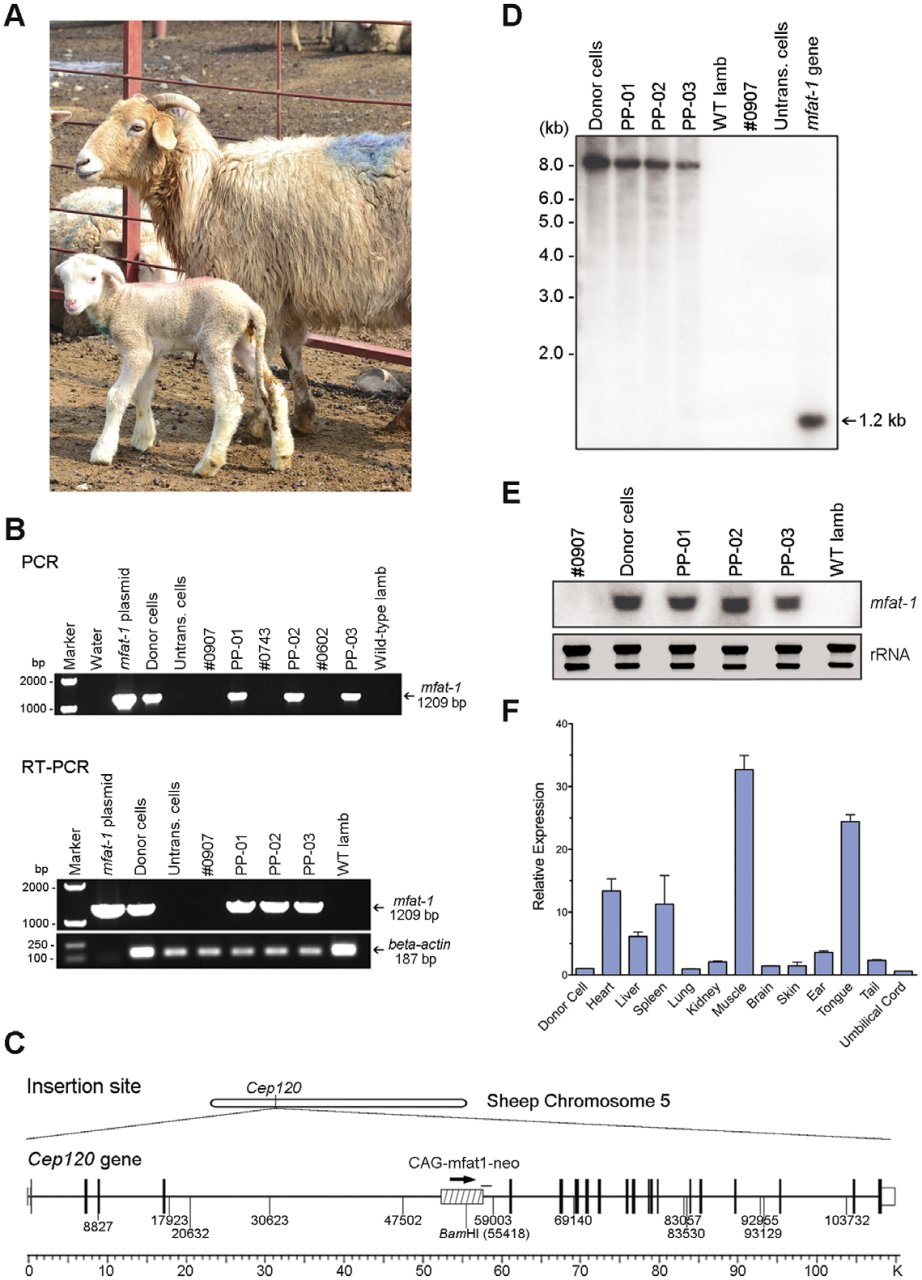
Production of transgenic lambs by handmade cloning. (**A**) The recipient #0907 and the transgenic lamb (PP-01). (**B**) Detection of the *mfat-1* gene in umbilical cord samples of three cloned lambs by PCR and RT-qPCR. (**C**) Insertion site of *mfat-1* vector in the sheep genome. Arrows indicate the *mfat-1* transcriptional direction, which is identical with the endogenous putative sheep *Cep120* gene. PCR fragment obtained in this study are shown by black bars. (**D**) Southern blot using the ^32^P-labled *mfat-1* specific sequence as a probe to hybridize the genomic DNA from the transgenic donor cells and the cloned lambs. The genomic DNA was digested with *Bam*HI before the gel electrophoresis. (**E**) Northern blot analysis. Total RNAs were loaded on each lane (15 µg per sample) and the coding region of *mfat-1* was used as a probe. Shown below is the gel electrophoresis of rRNA as control. (**F**) Quantitative PCR analysis of *mfat-1* expression in major tissues from the transgenic lambs (PP-02). Compared with the mRNA expression level normalized to the donor cell, the highest level of *mfat-1* expression was observed in transgenic muscle sample.

For the HMC procedure, approximately 3,740 (68%) out of 5,465 collected ovine oocytes were selected and utilized in the subsequent experiments. According to the established protocol, every two enucleated oocytes and a nuclear donor cell were combined by fusion to form one reconstructed embryo for the subsequent *in vitro* development. As a consequence, a total of 925 reconstructed embryos were produced and 82 (∼8.9%) of them successfully developed to the blastocyst stage after 7 days of *in vitro* culture (Table 2). A total of 53 blastocysts were surgically transplanted to uterus of 29 naturally cycling female recipients, with 1 to 2 blastocysts per recipient. Four pregnancies were detected and 3 live lambs were born either by caesarean section or natural delivery (Figure 2A and Table 3).

**Table 2:** Nuclear transfer efficiencies of the handmade cloning.

Donor cells		No. of reconstructed embryos	No. and (%) of cloned embryos developed to blastocysts	No. of blastocysts transferred	No. of recipients	No. and (%) ofpregnant	No. and (%) of lambs born
H-6-6	Passage 8	196	27 (13.8%)	13	6	0	0
	Passage 9	178	12 (6.7%)	9	5	2 (40.0%)	2 (22.2%)
	Passage 10	229	15 (6.6%)	11	6	0	0
	Passage 11	210	18 (8.6%)	13	7	1 (14.3%)	0
	Passage 12	112	10 (8.9%)	7	5	1 (20%)	1 (14.3%)
Total		925	82 (8.9±2.9%)	53	29	4 (13.8%)	3 (5.7%)

**Table 3:** Production of transgenic cloned lambs by HMC.

Lamb name	Donor cell	Recipient no.	Type of delivery (length of gestation)	Birth weight (kg)	Status
PP-01	H-6-6, Passage 9	#0907	Caesarean section (152 day)	5.74	Live (>7 months)
PP-02	H-6-6, Passage 9	#0743	Caesarean section (151 day)	3.86	Dead (2 days)
PP-03	H-6-6, Passage 12	#0602	Naturally (149 day)	5.24	Live (>7 months)

Genotype analysis using 13 ovine microsatellite markers demonstrated that all 3 transgenic sheep shared the same genotype to that of the nuclear donor cells (Table 4). The presence and function of *mfat-1* gene in the transgenic sheep were confirmed by PCR, RT-qPCR, Southern, Northern, and DNA sequencing (Figure 2 B, D, E). All 3 transgenic sheep are males as expected. The efficiency of nuclear transfer varied from 22.2% for the passage 9 to 14.3% for the passage 12 of the donor clonal cells, expressed as the number of live lambs per 100 blastocysts transplanted. The overall efficiency for production of live lambs (5.7%) was close to those reported previously [10], [18], [19]. The birth weight of the transgenic lambs ranged from 3.86 kg to 5.74 kg. The lambs PP-01 and PP-03 were healthy and behaved normally after the birth. The lamb PP-02, however, died within 48 hours after the birth with the lowest body weight among the three. The dead lamb was utilized as a representative sample to assess the levels of omega-3 in the muscle and other organs/tissues.

**Table 4:** Microsatellite analysis of three transgenic cloned lambs, donor cell, and recipient.

Loci	Lamb PP-01	Lamb PP-02	Lamb PP-03	Donor cell H-6-6	Recipient #0907	Recipient #0743
BMS460	122	122	122	122	122	122
	138	138	138	138	138	122
AE129	145	145	145	145	147	147
	147	147	147	147	149	149
MB009	142	142	142	142	142	142
	142	142	142	142	142	146
ETH3	103	103	103	103	95	103
	103	103	103	103	103	103
TGLA53	132	132	132	132	126	132
	132	132	132	132	126	132
INRA063	172	172	172	172	164	164
	178	178	178	178	172	172
ADCYC	246	246	246	246	246	244
	246	246	246	246	246	244
TGLA126	116	116	116	116	122	116
	116	116	116	116	126	116
MAF209	121	121	121	121	121	109
	127	127	127	127	121	121
TGLA122	146	146	146	146	158	138
	158	158	158	158	158	138
PZ963	253	253	253	253	227	209
	253	253	253	253	227	209
BMS2079	109	109	109	109	109	109
	109	109	109	109	115	109
BMS2104	147	147	147	147	147	147
	147	147	147	147	153	147

Insertion site analysis demonstrated that a single copy of the *mfat-1* was integrated into the sheep genome on chromosome 5, located within the 5^th^ intron of a functional gene *Centrosomal protein of 120* *kDa* (*Cep120,* Figure 2C). DNA sequencing of a 1.2 kb PCR fragment across the 3′-end of *mfat-1* and the neighbor host genome showed a 99% sequence homology to exons 5 and 6 of *Cep120* (Figure 2C). The transcriptional orientation of the transgene *mfat-1* was identical to that of the host gene. We found that the insertion of the *mfat-1* showed no significant interfering effect on the function of *Cep120*, which is involved in centriole assembly and is essential for cell division [20], [21]. The remaining 2 transgenic lambs showed no obvious sign of neurogenic or other defects up to the date.

Single-copy insertion of the *mfat-1* to ovine genome was cross-verified by the *Bam*HI restriction enzyme analysis. Southern blot revealed a single target band sized ∼8.0 kb in *Bam*HI digested DNA only from the transgenic lambs (Figure 2D). This result was consistent with the previous analysis in that the DNA fragment digested with *Bam*HI was expected to be 7916 bps in length (Figure 2C). The possibility of tandem insertion of multiple *mfat-1* copy was excluded, because the digested *Bam*HI band from the transgenic sheep failed to show a predictive 5.2 kb fragment, a band representing multiple copies of the *mfat-1* with the maximum size of 5.2 kb.

**Figure 3:**
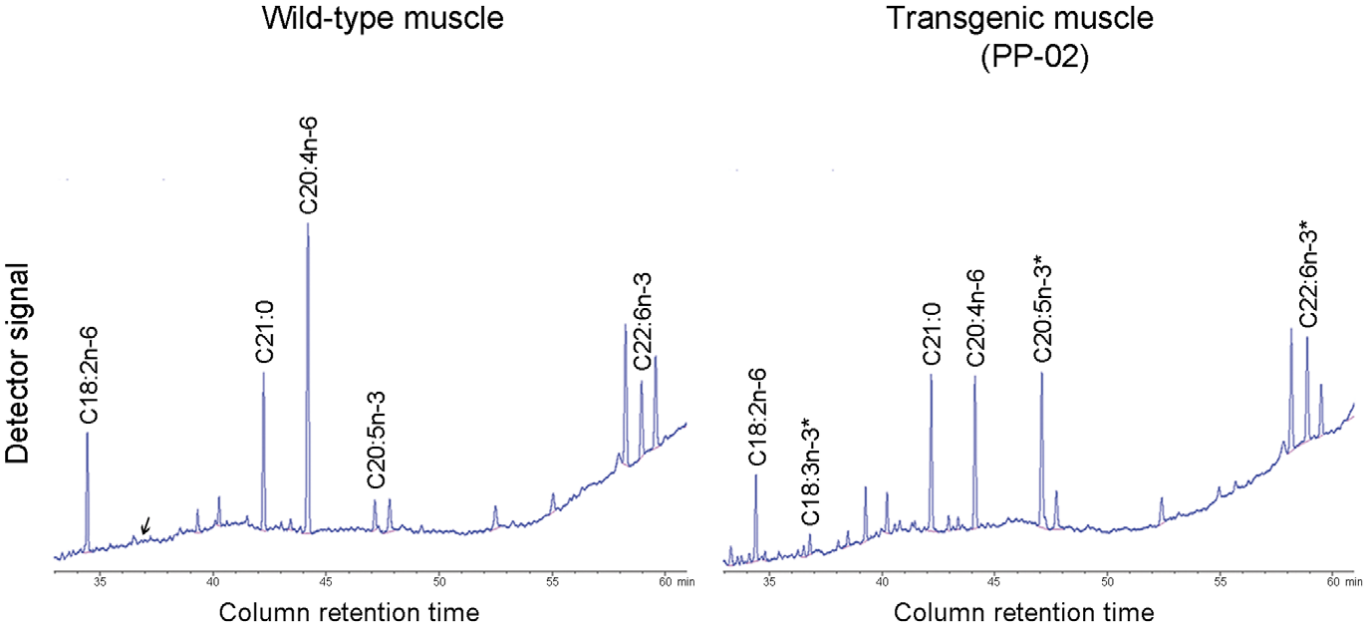
Partial gas chromatograph of fatty acids in muscle sample of *mfat-1* transgenic (PP-02) and the control lamb. Fatty acid methyl esters were quantified using a fully automated 6890 Network GC System with an Agilent J&W fused-silica DB-23 capillary column. The peaks were identified by comparison with the internal fatty acid standards, and area percentage for all of resolved peaks was analyzed using GC ChemStation software. Compared with the wild-type control (left), the level of n−6 polyunsaturated acids in the transgenic muscle (right) are significantly lower, whereas n−3 fatty acids are abundant.

Analyses of fatty acid composition showed that the omega-3 (n−3) peaks in the umbilical cords of all three transgenic lambs were significantly higher than those in the age-matched wild type controls (data not shown). The results was cross-verified by systematic analysis of the fatty acid in major tissues of the lamb PP-02, which showed a two-fold reduction of n−6/n−3 ratio (2.63 to 1.24, *p*<0.01) in muscles compared to the wild-type lamb (Figure 3, Table 5). The lower ratio of n−6/n−3 was also found in other major tissues (*p*<0.05, Table 5). Consistent with these results, the level of *mfat-1* mRNA varied among different tissues, with the highest expression found in the skeletal muscle (Figure 2F). More importantly, the total n−3 fatty acids (a-linolenic acid, eicosapentaenoic acid (EPA), and docosahexaenoic acid (DHA)) constituted approximately 6.2% of total muscle fat in the transgenic lamb, significantly higher than those in the control lambs, which was 3.5% on average (*p*<0.01). All of these indicated that the *mfat-1* gene was functional in the transgenic lambs. Compared to the earlier studies of transgenic animals expressing the n−3 fatty acid desaturase gene [22], [23], our results help to provide additional evidence on the feasibility of transgenic animals.

**Table 5:** Comparison of n−6/n−3 ratios between wild-type and *mfat-1* transgenic lamb.

Organs or Tissues	*n*−6/*n*−3 Ratio^a^	
	Wild-type	Transgenic
Heart	2.28±0.01	0.93±0.02**
Liver	1.56±0.05	0.66±0.04**
Spleen	3.04±0.23	1.50±0.16**
Lung	2.66±0.46	1.40±0.12*
Kidney	2.45±0.05	1.36±0.15**
Brain	0.42±0.01	0.32±0.01**
Ear	2.89±0.02	1.18±0.25**
Tongue	1.66±0.13	1.25±0.02*
Tail	4.60±1.14	2.08±0.63*
Muscle	2.63±0.05	1.24±0.06**

Ratio of n−6/n−3 fatty acid was calculated from n−6 fatty acids [linoleic acid (LA, 18∶2n−6) and arachidonic acid (AA, 20∶4n−6)] versus n−3 fatty acids [α-linolenic acid (ALA, 18∶3n−3), eicosapentaenoic acid (EPA, 20∶5n−3), and docosahexaenoic acid (DHA, 22∶6n−3)]. Each value represents the mean ± standard deviation from three replicated sample measurements of each tissue.

a Statistical analysis using the two tailed student *t*-test. Significant differences between the wild-type and the transgenic lamb samples were marked (*P<0.05; **P<0.01).

It was not escaped from our attention that, in contrast to the SCNT, the procedure of HMC introduced an obvious heteroplasmy into the transgenic animals, mitochondrial DNAs in particular. While the potential interactions and the long-term consequences of such heteroplasmy remain to be determined, it seems that the developmental reprogramming driven by the enucleated oocytes was not significantly affected. Although certain concern was raised regarding the three origins of mitochondrial DNAs in the developing fetus from the cells of different sheep, no evidence of the negative effect to the transgenic animal is currently available [24]. The two remaining HMC sheep in our study showed normal growth and development up to the date (Figure S1), consistent with those of the HMC pigs [3], [15].

Over the past decade the nematode *fat-1* has been successfully introduced into mice [8], [25], pigs [2], [3] and cattle [9]. The *fat-1* mice produced by pronuclei microinjection have possessed good value for the production of n−3 PUFAs [8], [25], particularly for DHA and docosapentaenoic acid (DPA) in transgenic *sFat-1* mice [25]. In the transgenic pig, Lai et al. (2006) reported that there was a fivefold reduction of the n−6/n−3 ratio in tail tissues of *hfat-1* transgenic piglets. The other tissues from transgenic piglets also showed a substantially lower n−6/n−3 ratio [2]. Wu et al. successfully cloned transgenic cow expressing *fat-1* gene by traditional SCNT, with increased levels of n−3 PUFAs in the ear tissue, and reduced ratio of n−6/n−3 in the milk [9]. Our previous results also showed that nematode *mfat-1* could effectively lower the n−6/n−3 ratio in muscle and other major organs of the transgenic pig [3].

In summary, we used the modified HMC procedure to generate a group of transgenic sheep carrying a functional nematode *mfat-1* gene, and the resulting sheep were rich in omega-3 fatty acids in the muscles and other organ/tissues. Our study demonstrated once again that the alternative HMC is helpful to accelerate the speed of transgenic animal research with cost efficiency and easy in operation. With an increasing demand for dietary intake of omega-3 for a better quality of our life via disease prevention, generation of transgenic animals rich in omega-3 could be an attractive alternative not only for human health, also for environmental conservation.

## Materials and Methods

All the chemical reagents used in this research were obtained from Sigma-Aldrich Chemical (St Louis, USA) except for otherwise indicated.

### Ethics Statement

This study was carried out following the recommendations in the Guide for the Care and Use of Laboratory Animals of the Chinese Academy of Sciences. Procedures for all of the animal experiments were approved in advance by the Animal Care and Ethics Committee of the Institute of Genetics and Developmental Biology, Chinese Academy of Sciences.

### Ovine Primary Fibroblasts Cell Culture

Punch collected ear tissue from a Chinese merino male sheep was washed twice with Ca^2+^- and Mg^2+^-free PBS (DPBS), minced with a surgical blade on a 10-cm culture dish (Becton Dickinson, Lincoln Park, USA) and then dissociated with 0.25% (v/v) trypsin-EDTA (GIBCO, New York, USA) containing Dulbecco’s modified Eagle’s medium (DMEM, GIBCO) at 37°C. Cell pellets after centrifuging were seeded onto a 35 mm culture dish and cultured for 8–10 days in DMEM supplemented with 10% (v/v) fetal bovine serum (FBS, HyClone, Logan, USA) at 37°C and 5% CO_2_. The construction of *mfat-1* expression vector CAG-mfat1-neo and the transfection were performed as described previously [3], [26]. Cells in each well were trypsinized and seed onto a 10-cm culture dish containing 450 µg/mL Geneticin (Invitrogen, USA) for 24 hrs after the transfection. Drug selection was conducted for 2 weeks with a medium change per 3 days and the Geneticin-resistant colonies were isolated. Each colony was transferred to a 6-well plate for expansion. The resulting clones were frozen at −80°C overnight and stored in liquid nitrogen.

### Analysis of *mfat-1* in the Ovine Cell Clones

Geneticin selected cells were analyzed by PCR and RT-PCR as described previously [3]. Quantitative PCR (qPCR) was performed with sequence-specific primer pairs for *mfat-1* and *beta-actin,* respectively. Reagents of Power SYBR® Green PCR Master Mix were used for the PCR amplification in triplicates under the following thermal program: 10 min at 95°C, 40 cycles of 95°C for 15 sec, 60°C for 25 sec, and 72°C for 20 sec.

The *mfat-1* positive cells were cultured in medium supplemented with 10 µM 18∶2n−6 and 15 µM 20∶4n−6 prior to fatty acid analysis. Cells were collected in a glass methylation tube for fatty acid analysis at 80% confluence. The fatty acid compositions of total cellular lipids were analyzed for both of the transgenic and control clones using the gas chromatography as previously described [27]. Fatty acid methyl esters were quantified using a fully automated 6890 Network GC System (Agilent Technologies, Palo Alto, USA) with an Agilent J&W fused-silica DB-23 capillary column. The injector and detector were maintained at 260°C and 270°C, respectively. The oven program was maintained initially at 180°C for 10 min, then ramped to 200°C at 4°C/min and held for 15 min, finally ramped to 230°C at 10°C/min and maintained for 6 min. Carrier gas-flow rate was maintained at a constant rate of 2.0 mL/min throughput. The peaks were identified by comparison with the internal fatty acid standards, and area percentage for all of resolved peaks was analyzed using the GC ChemStation software (Agilent Technologies, USA).

### Procedure Outline for the Handmade Cloning

Sheep ovaries were collected at a local slaughterhouse of Xinjiang Hualing Industry and Trade (Group) Co. Ltd., with the authorization from the business administration for the collection and usage of ovary tissues on the transgenic research. The fresh ovary tissues were transferred to a portable incubator for processing within 120 min and the oocytes were isolated by the ovarian slicing method described previously [28]. Prior to slicing, ovaries were washed in phosphate buffered saline (PBS), and then placed in a Petri dish and covered with Hepes-buffered TCM-199 (GIBCO, USA) containing 60 IU/mL heparin. The compact cumulus-oocytes complexes (COCs) were selected and washed twice in Hepes-buffered TCM-199. Each group of 50 COCs was matured in 400 µL bicarbonate-buffered TCM-199 supplemented with 10% (V:V) fetal bovine serum (FBS), 2 mM L-Glutamine, 0.3 mM sodium pyruvate, 0.1 mM cysteine, 5 µg/mL follicle stimulating hormone, 5 µg/mL luteinizing hormone, 1 µg/mL β-Estradiol in an incubator maintained at 38.6°C with humidified air and 5% CO_2_.

The HMC was performed essentially as previously described [13], [15], with minor modifications to adapt for the ovine nuclear transferring. Briefly, at 21–22 h after the starting point of maturation, the cumulus cells were removed by repeated pipetting in 1 mg/mL hyaluronidase in Hepes-buffered TCM-199. All the remaining manipulations were performed on a hotplate adjusted to 30°C. Zonae pellucidae were partially digested with 8 mg/mL pronase solution dissolved in T33 (T for Hepes-buffered TCM 199 medium, and the number for the percentage of calf serum supplement), then washed quickly in T2 and T20 drops. The oocytes with softened zonae pellucidae were lined up in T20 drops supplemented with 2.5 µg/mL cytochalasin B (CB), and were enucleated by oriented bisection with an ultra sharp microblade (AB Technology, Pullman, USA) under a stereomicroscope. Approximately 1/3−1/2 cytoplasm close to the polar body was removed manually, and the remaining putative cytoplast were washed twice in T2 drops and collected in a T10 drop. The nuclear donor cells were trypsinized and kept in T2. Fusion was performed in two steps where the second step included the initiation of activation. For the first step, half of the available cytoplasts were transferred into 1 mg/mL of phytohaemagglutinin (PHA; ICN Pharmaceuticals, Australia) dissolved in T0, and then each one was quickly dropped over a single fibroblast. After attachment, cytoplast-fibroblast pairs were equilibrated in a fusion medium (0.3 M mannitol and 0.01% polyvinyl alcohol). Using an alternative current (AC) of 4 V (CF-150/B fusion machine; BLS, Budapest, Hungary), cell pairs were aligned to the wire of a fusion chamber (BTX microslide 0.5 mm fusion chamber, model 450; BTX, SanDiego, USA) with the somatic cells farthest from the wire, then fused with a single direct current (DC) of 1.3 kV/cm for 10 µsec. After the pulse, cytoplast-fibroblast pairs were incubated in T10 drops to examine whether or not the fusion had occurred. Approximately 1 h after the first fusion, each pair was fused with the second cytoplasts and activated simultaneously in activation medium (0.3 M mannitol, 0.1 mM MgSO4, 0.1 mM CaCl_2_ and 0.01% polyvinyl alcohol) with a double DC pulse of 0.9 kV/cm, each pulse for 15 µsec and 1 sec apart. When fusion had been observed in T10 drops, the reconstructed embryos were first incubated in 40 µL Hepes-buffered TCM 199 medium containing 5 µM Ionomycin for 5 min at 38.6°C. After subsequent washing in culture medium 3–5 times, reconstructed embryos were incubated in culture medium containing 2 mM 6-dimethylamino-purine (6-DMAP) for 4 h at 38.6°C in 5% CO_2_ with maximum humidity, then washed thoroughly with IVC medium for culture.

The reconstructed embryos were *in vitro* cultured in wells of a Nunc 4-well dish in 400 µL SOFaaci medium [29] supplemented with 4 mg/mL BSA and covered with oil. Zona-free embryos produced from the HMC were cultured in modified WOWs system [30], [31] at 38.6°C in 5% CO_2_ with saturated humidity. Cleavage and blastocyst formation of the reconstructed embryos were monitored during the *in vitro* culture for 7 days.

### Embryo Transfer and Pregnancy Diagnosis

The healthy ewes from a local Xinjiang sheep breed aged 12–18 months were selected as the embryo-transfer recipients. The blastocysts with clearly visible inner cell mass were surgically transplanted into the uterine horns of a naturally cycling ewe on 6 or 7 days of standing estrus by a certified veterinarian. Pregnancies were diagnosed by ultrasonography on day 45 after the surgical embryo transfer.

### Screening of the Transgenic Sheep

Umbilical cord from each newborn lamb was collected and the genomic DNA was isolated for the genotyping and genetic screening. Presence of *mfat-1* gene in each DNA sample was testified by SP-PCR and Southern blot. The expression of *mfat-1* mRNA was evaluated by RT-PCR and Northern blot.

Comparative genotype analyses were performed for each of the newborn lambs, the recipients, and the donor fibroblasts using a set of microsatellite markers. Thirteen polymorphic microsatellite loci (BMS460, AE129, MB009, ETH3, TGLA53, INRA063, ADCYC, TGLA126, MAF209, TGLA122, PZ963, BMS2079, BMS2104) located on different ovine chromosomes were amplified by 3-color multiplex PCR and the products were analyzed on a 3130XL Genetic Analyzer (Applied Biosystems) with GeneMapper ID Software v3.2 (Applied Biosystems, USA).

For Southern blot analysis, approximately 20 µg of genomic DNA was digested with *Bam*HI for agarose gel electrophoresis. The probes were ^32^P -labeled PCR fragments of *mfat-1* sequence, generated using the Random Prime Labeling System Redi PrimeTMII (GE Healthcare, Piscataway, USA). Northern blot analysis was performed as described previously [32]. About 15 µg of total RNA were loaded per lane, and the coding region of *mfat-1* was used as a probe.

To identify the insertion site, the genomic DNA flanking inserted *mfat-1* was amplified by a thermal asymmetric interlaced PCR (TAIL-PCR) using Genome Walking Kit (TaKaRa, Japan). The specific primers (CAG4823-1F: 5′-cctcgtgctttacggtatcg-3′; CAG-4939-2F: 5′-caacctgccatcacgagatt-3′ and CAG5044-3F: 5′-tctcatgctggagttcttcg-3′) were designed with their melting temperatures sufficiently high to ensure a maximum thermal asymmetric priming and to ensure the cloning of genomic DNA flanking the *mfat-1* gene. The tertiary TAIL-PCR products were purified, cloned into pMD18-T (TaKaRa, Japan), and sequenced. The BLAST homology searches and the sequence comparisons with the draft sheep genome assembly OARv2.0 (International Sheep Genomics Consortium) were performed to identify the site of *mfat-1* insertion at the level of nucleotide sequences.

To evaluate the functional activity of desaturase encoded by nematode *mfat-1* gene, the tissues from one of the transgenic lambs (PP-02) was utilized as representative samples for fatty acid analyses. The major organ/tissue samples (heart, liver, spleen, lung, kidney, tongue, ear, muscle, brain, and tail) were collected from both transgenic and age-matched wild-type lamb, and the fatty acid analysis was performed as described previously. Briefly, the oven program of the gas chromatography instrument was maintained initially at 100°C for 5 min, then ramped to 180°C at 6°C/min and held for 10 min, then ramped to 205°C at 2°C/min and held for 10 min, finally ramped to 225°C at 2°C/min and maintained for 7 min. The ratio of n-6/n-3 from each organ/tissue was used for comparison between the transgenic and the wild-type lambs.

### Statistical Analysis

Statistical analysis was performed using a two-tailed Student’s *t*-test, and p-values <0.05 were considered statistically significant.

## Supporting Information

Figure S1 **Image of** ***mfat-1*** **transgenic lambs of approximately 75 days (PP-01) and 100 days (PP-03) after the birth.** The lamb (PP-02) was used for n−3 fatty acid composition analyses of major organ/tissues approximately 3 days after the birth, due to obvious weakness at the time of birth.
(TIF)
