# Supplementary figures and images for: Handmade Cloned Transgenic Sheep Rich in Omega-3 Fatty Acids
Source: PLoS One. 2013 Feb 20;8(2):e55941. doi: 10.1371/journal.pone.0055941 (PMC3577796; doi:10.1371/journal.pone.0055941)

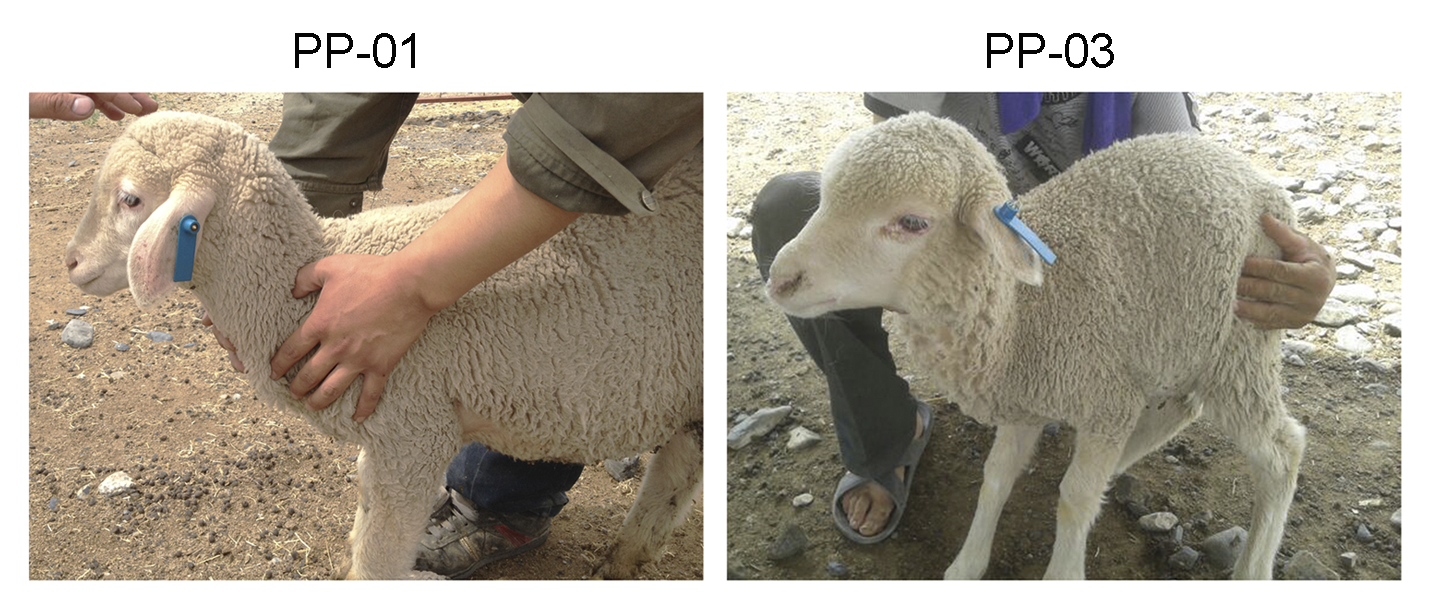

Supplement: Figure S1 — Image of mfat-1 transgenic lambs of approximately 75 days (PP-01) and 100 days (PP-03) after the birth. The lamb (PP-02) was used for n−3 fatty acid composition analyses of major organ/tissues approximately 3 days after the birth, due to obvious weakness at the time of birth. (TIF) [file pone.0055941.s001.tif]
